# On We Go, with Hope: Remembering Our Founding Editor-in-Chief, Dr. F. Douglas Scutchfield

**DOI:** 10.13023/jah.0402.02

**Published:** 2022-07-01

**Authors:** Rachel E. Dixon, Randolph F. Wykoff

**Affiliations:** University of Kentucky, managingeditor.jah@gmail.com; East Tennessee State University

**Keywords:** Appalachia, legacy, journal editing, editorial board, obituary

## Abstract

With the passing of our founding Editor-in-Chief, Dr. F. Douglas Scutchfield, the Journal of Appalachian Health team reflects on a life well lived and a monumental public legacy left behind. We thank “Scutch” for enriching the lives of so many, and we commit to growing the Journal in his honor.

You do not need to know precisely what is happening, or exactly where it is all going. What you need is to recognize the possibilities and challenges offered by the present moment, and to embrace them with courage, faith and hope.

— Thomas Merton, *Conjectures of a Guilty Bystander*

To say that F. Douglas Scutchfield, MD—known to many of us warmly, and quite simply, as “Scutch”—was a Renaissance man with reverberations far beyond public health seems insufficient to capture all that he was and *is*. A humble recipient of health and humanities accolades alike, Scutch was not just an observer of life’s complexities but a truly active participant in shaping the systems and minds around him. He didn’t just *have* dreams; he “gave ’em wings” (as another of our favorite Appalachians often advocates).^1^

One of those dreams was this publication—a *Journal of Appalachian Health* dedicated to investigating, chronicling, and advancing the well-being of the people who call Appalachia’s hills home.

Scutch was himself a native of Appalachia, born amidst the Second World War in Wheelwright, Kentucky. With just over 500 residents at the last Census,^2^ Wheelwright epitomizes small-town life. That Scutch was born in a small town sharing its name with a wheel-repairer seems particularly apt—after all, it was his tireless advocacy that got the Journal not just moving, but cruising. Shortly before Dr. Scutchfield’s untimely death last month, the editorial team learned that our publication would be indexed on PubMed Central—an honor reserved for top journals in the biomedical and life sciences. This monumental accomplishment is a direct result of Scutch’s ability to combine strategic insight and technical expertise with the patience and charisma needed to nurture a journal from the whisper of an idea to a well-read success.

This journal is Scutch’s legacy—a tour de force in a career dedicated to bettering the lives of those around him. It caps off an impressive portfolio which took Scutch from the hills of Floyd County to Chicago, Atlanta, Tuscaloosa, San Diego, Israel, and home to Kentucky again. Through his many experiences— which included practicing family medicine, founding schools of public health,^3^ consulting for national governments^4^, and serving in the US Epidemic Intelligence Service, among other activities—Scutch assembled a network of public health leaders across the globe, and he made key advancements in our understanding of the social determinants of health.

As this new era for the Journal dawns, we are committed to carrying Scutch’s work forward. Dr. Scutchfield himself wrote in our last issue that there are new faces aboard to help captain the *Journal of Appalachian Health*.^5^ With this issue, one of us (RW) assumes the mantle of Editor in Chief, and another (RD) assumes the role of Managing Editor. We are fortunate to be supported by phenomenally engaged editorial and advisory boards and a growing number of colleagues and collaborators who are coming to recognize the Journal as an ideal place to share thoughts and ideas on the future of health and well-being in Appalachia. We look to build on the partnerships Scutch initiated and introduce new initiatives to expand thinking around health and geography and to uproot the myriad inequities—based on poverty, place, race, ethnicity, sexual orientation, gender identity, and other factors—that Scutch worked tirelessly to address in Appalachia.

The present moment presents unparalleled challenges—both in the transition of our Journal and in society at large—but we move forward with courage and continued faith in the power of evidence-based public health research to change lives for the better. This was the task that Scutch recognized and committed himself to. Perhaps more remarkable than his mind for public health was his ability to see possibility in even the bleakest situations, and we want to capture that spirit. So as he would write, “on we go!”, with hope.
